# Sleep Quality, Insomnia Symptoms, and Perceived Fatigue in Relation to Countermovement Jump Performance Across Repeated Trials in Resistance-Trained Adults

**DOI:** 10.3390/healthcare14162543

**Published:** 2026-08-14

**Authors:** Dawid Koźlenia, Marek Popowczak

**Affiliations:** Faculty of Physical Education and Sport, Wroclaw University of Health and Sport Sciences, al. I.J. Paderewskiego 35, 51-612 Wrocław, Poland; marek.popwczak@awf.wroc.pl

**Keywords:** within-session performance, jump performance, countermovement jump, sleep disturbance, perceived fatigue, resistance training

## Abstract

**Objectives:** This study examined the associations of habitual sleep quality, insomnia symptoms, and perceived fatigue with countermovement jump (CMJ) performance across repeated trials in resistance-trained adults. **Methods:** A total of 118 resistance-trained adults (90 men and 28 women) completed the Pittsburgh Sleep Quality Index (PSQI), Insomnia Severity Index (ISI), and Fatigue Severity Scale (FSS). Participants performed six maximal CMJ trials at 0, 2, 4, 6, 8, and 10 min. Mean and best CMJ height, within-participant coefficient of variation, first-to-last change, and individual performance slope were calculated. Linear mixed-effects models and adjusted regression analyses were applied. **Results:** PSQI was not associated with mean CMJ height, best CMJ height, or within-participant variability (all *p* > 0.05). However, a significant PSQI × time interaction was observed (B = −0.061 cm/min per 1-SD higher PSQI, 95% CI −0.099 to −0.023; *p* = 0.002; q = 0.005), indicating that poorer sleep quality was associated with a smaller increase in CMJ height across repeated trials. Exploratory participant-level analyses derived from the same six CMJ measurements showed associations in the same direction for first-to-last change and individual CMJ slope. These associations remained significant after correction within the six trajectory-focused analyses but not after correction across all 18 participant-level regressions. ISI and FSS were not associated with CMJ trajectory or variability (all *p* > 0.05). **Conclusions:** Poorer habitual sleep quality was not associated with maximal or average CMJ performance, but was related to an attenuated improvement across repeated maximal CMJ trials. The observed association was small, and its practical significance remains uncertain. The within-session CMJ trajectory should therefore be considered exploratory rather than a validated indicator of recovery or neuromuscular readiness.

## 1. Introduction

Fatigue and recovery are important determinants of an individual’s capacity to participate safely and effectively in physical activity. In athletic and recreationally active populations, insufficient recovery may affect force production, motor control, movement efficiency, and the ability to maintain physical performance across repeated efforts. Monitoring recovery-related factors is therefore an important component of training management, as individuals exposed to similar external workloads may demonstrate different functional responses depending on their current psychophysiological state. Sleep is one of the principal processes supporting recovery, training adaptation, and neuromuscular function. Adequate sleep contributes to endocrine regulation, immune function, tissue repair, cognitive processing, and restoration of the nervous system, whereas insufficient or disturbed sleep may compromise several of these processes [1,2,3].

Athletes and resistance-trained individuals may be particularly susceptible to suboptimal sleep because of demanding training schedules, early-morning or late-evening sessions, competition-related stress, travel, and the need to combine exercise with occupational, educational, and social responsibilities [1,2]. Sleep disturbances may therefore contribute to incomplete recovery and increased perceived fatigue, with potential consequences for subsequent physical functioning. Sleep-extension and sleep-hygiene interventions have been associated with improvements in selected aspects of recovery and athletic performance, although the magnitude of these effects varies according to the sleep intervention and the performance outcome examined [4].

Experimental evidence generally indicates that acute sleep loss can impair exercise performance, perceived exertion, vigilance, coordination, and motor control [5,6]. However, the effects on power performance are less consistent than those observed for prolonged, intermittent, or cognitively demanding exercise. Li and Mei [7] reported that a single night of partial sleep deprivation impaired anaerobic performance and reactive agility but did not significantly affect countermovement jump (CMJ) height. Conversely, Mah et al. [8] found that three consecutive nights of restricted sleep reduced maximal jump performance and increased lower-extremity coordination variability in elite athletes. These findings suggest that inadequate sleep may not uniformly suppress the ability to produce one maximal effort. Instead, its functional consequences may become more apparent through alterations in movement organization, performance reproducibility, or the ability to maintain neuromuscular output across successive attempts.

Most previous studies have investigated experimentally induced sleep restriction or deprivation. Although these designs provide evidence regarding the effects of acute sleep loss, they may not fully represent the habitual sleep disturbances and fatigue experienced under typical training and daily life conditions. Observational evidence relating habitual recovery-related characteristics to physical function remains comparatively limited. In professional rugby union athletes, longer sleep duration during preseason training was associated with more favorable changes in aerobic fitness and body composition, although associations with strength and speed were less evident [9]. Cabarkapa et al. [10] additionally reported relationships between poorer Pittsburgh Sleep Quality Index (PSQI) scores and selected concentric force and power characteristics of the CMJ in semi-professional basketball players. However, research has largely focused on isolated or best-performance outcomes, while considerably less is known about whether habitual sleep disturbances and perceived fatigue are related to the maintenance and reproducibility of performance across repeated maximal trials.

This distinction may be relevant when characterizing CMJ performance across repeated trials. A single best result primarily reflects the highest performance capacity demonstrated under the testing conditions. In contrast, the mean response, within-participant variability, and change across successive trials may provide complementary information about within-session performance patterns. Repeated maximal CMJ trials separated by standardized recovery intervals should not be interpreted as a direct test of fatigue resistance or repeated-power ability. Nevertheless, analysing their trajectory may help identify subtle functional differences associated with recovery-related characteristics that are not evident from a single maximal attempt. In the present protocol, which followed five progressive practice CMJs and did not include a fatigue-inducing task, systematic changes across trials could also reflect continued task-specific warm-up, learning, increased arousal, or technical optimization. The trajectory was therefore examined as an exploratory within-session outcome without attributing it to a specific physiological or technical mechanism. The CMJ is widely used to assess lower-body power performance and neuromuscular status because it requires the rapid integration of force production, movement velocity, and stretch–shortening cycle function. Sleep quality, insomnia symptoms, and perceived fatigue represent related but distinct aspects of recovery status. The PSQI provides a multidimensional assessment of habitual sleep quality and sleep-related disturbances over the preceding month [11]. The Insomnia Severity Index (ISI) evaluates the perceived severity of difficulties initiating or maintaining sleep, dissatisfaction with sleep, and the daytime consequences and distress associated with these symptoms [12]. The Fatigue Severity Scale (FSS), in turn, assesses the perceived impact of fatigue on motivation, physical functioning, and everyday activities [13]. Examining these measures concurrently may provide a broader perspective on recovery because sleep disturbances do not necessarily translate directly into daytime fatigue, and individuals reporting similar sleep quality may differ in their functional responses.

Therefore, the primary aim of this study was to examine the associations of habitual sleep quality, insomnia symptoms, and perceived fatigue with CMJ performance level, within-participant variability, and within-session performance trajectory across repeated maximal trials in resistance-trained adults. Specifically, the study investigated whether higher PSQI, ISI, and FSS scores were associated with lower mean and best CMJ performance, greater within-participant variability, and a less favourable performance trajectory across successive trials. We hypothesized that higher PSQI, ISI, and FSS scores would be associated with lower mean and best CMJ height, greater within-participant variability, and a less favourable performance trajectory across successive trials, with more consistent associations expected for trajectory and variability than for best performance.

## 2. Materials and Methods

### 2.1. Study Design

This cross-sectional observational study examined the associations of habitual sleep quality, insomnia symptoms, and perceived fatigue with countermovement jump (CMJ) performance in resistance-trained adults. During a single laboratory session, participants completed the PSQI, ISI, and FSS questionnaires, underwent anthropometric assessment, performed a standardized warm-up, and completed six maximal CMJ trials. The CMJ trials were performed at 0, 2, 4, 6, 8, and 10 min, with 2 min recovery intervals between attempts. Six evenly spaced trials were selected to provide repeated observations across a standardized 10 min period, allowing the exploratory estimation of within-session performance patterns while limiting the total number of maximal efforts. Previous CMJ reliability protocols have used approximately 1 min intervals between maximal attempts [14,15]. The 2 min interval used in the present study was selected as a conservative standardized design choice intended to reduce immediate carryover and fatigue accumulation between individual CMJs. It was not intended to assess recovery kinetics, and complete physiological recovery was neither assumed nor verified. The repeated measurements were used to assess mean and best CMJ height, within-participant variability, first-to-last change, and the individual performance trajectory across the session. No sleep manipulation or fatigue-inducing exercise protocol was applied. The study was conducted in accordance with the Declaration of Helsinki and reported according to the STROBE recommendations [16].

### 2.2. Participants

A total of 118 resistance-trained adults, including 90 men and 28 women, participated in the study. Participants were 18–32 years of age and were recruited from the local university and resistance-training community through direct contact and voluntary response to study announcements. Participants reported 5.1 ± 2.7 years of continuous resistance-training experience, 3.8 ± 1.3 resistance-training sessions per week, and a weekly training volume of 290 ± 102 min·week^−1^. Participants were eligible if they were at least 18 years old, had engaged in structured resistance training at least twice per week for a minimum of six months, were familiar with maximal vertical jumping, and were able to perform the countermovement jump protocol safely. The exclusion criteria included a current lower-limb musculoskeletal injury, pain limiting maximal jumping, a neurological, cardiovascular, or other medical condition contraindicating maximal physical effort, the use of medication that could substantially affect sleep or neuromuscular performance, and failure to comply with the pre-test instructions. Only participants who completed all questionnaires and the full CMJ testing protocol were included in the analysis. Participants were instructed to maintain their habitual sleep and dietary patterns before testing and not to deliberately alter their usual sleep duration. They were asked to avoid strenuous physical exercise and alcohol consumption for 24 h before the session, caffeine-containing products for at least 6 h, and food intake for at least 3 h before testing. Participants were permitted to consume water and were asked to arrive normally hydrated. They were also instructed not to introduce any new dietary supplements or medications before the assessment. Testing was conducted under standardized laboratory conditions, and the time of testing was 9–12 a.m. Before participation, all individuals received verbal and written information regarding the purpose and procedures of the study, potential risks, confidentiality of the collected data, and their right to withdraw at any time without providing a reason. Written informed consent was obtained from all participants before data collection. The study was conducted in accordance with the Declaration of Helsinki and was approved by the Ethics Committee of the Wroclaw University of Health and Sport Sciences (approval no. 06/2023; 31 March 2023).

### 2.3. Sample Size

An a priori sample-size calculation was performed using G*Power version 3.1.9.2 (F tests: linear multiple regression, fixed model, R^2^ increase). The calculation was based on the planned participant-level regression analysis of individual CMJ slope, which was used as a participant-level approximation of the intended PSQI × time association because G*Power does not directly implement the mixed-effects model used to analyse the complete repeated-measures structure. In the absence of directly comparable previous estimates, an incremental effect size of f^2^ = 0.08 was selected before data analysis as a pragmatic small-to-moderate planning assumption. This value lies between the conventional small and medium effect-size benchmarks and reflects the expectation that PSQI would explain a modest additional proportion of the variance in CMJ slope beyond sex, age, and BMI. The calculation additionally assumed α = 0.05, 80% statistical power, one tested predictor (PSQI score), and four total predictors (PSQI score, sex, age, and BMI). The required minimum sample size was 101 participants. The final analytical sample of 118 participants exceeded this requirement.

### 2.4. Anthropometric Assessment

Anthropometric measurements were performed according to standardized procedures recommended by the International Society for the Advancement of Kinanthropometry [17]. Body height was measured barefoot to the nearest 0.1 cm using a GPM anthropometer (GPM Anthropological Instruments, DKSH Ltd., Zürich, Switzerland). Participants stood in an upright position with their heels together, arms relaxed alongside the body, and head aligned in the Frankfort horizontal plane.

Body mass was measured to the nearest 0.1 kg using the integrated scale of the InBody 230 analyser (InBody Co., Ltd., Cerritos, CA, USA) [18]. Participants were assessed barefoot, wearing light clothing, after emptying their bladder. They were instructed to refrain from food intake and strenuous physical activity for at least 3 h before the measurement and to maintain normal hydration.

Before each testing session, the measurement devices were inspected, levelled, and zeroed according to the manufacturers’ recommendations. The same equipment and standardized measurement procedures were used for all participants. Only body mass recorded by the InBody 230 analyser was used in the present study; bioelectrical-impedance-derived body-composition variables were not included in the analyses.

Body mass index was calculated as follows:

BMI (kg/m^2^) = body mass (kg)/[body height (m)]^2^

### 2.5. Sleep Quality, Insomnia Symptoms, and Perceived Fatigue

Participants completed Polish-language versions of the Pittsburgh Sleep Quality Index (PSQI), Insomnia Severity Index (ISI), and Fatigue Severity Scale (FSS). The questionnaires were administered in paper form in a quiet laboratory environment before the anthropometric measurements, standardized warm-up, and CMJ assessment. Participants completed the questionnaires independently and were instructed to select the response that best reflected their experiences during the time period specified for each instrument. A researcher remained available to clarify the meaning of the instructions when necessary but did not influence the participants’ responses.

#### 2.5.1. Pittsburgh Sleep Quality Index

Habitual sleep quality over the preceding month was assessed using the PSQI [11]. The questionnaire consists of 19 self-reported items used to derive seven component scores: subjective sleep quality, sleep latency, sleep duration, habitual sleep efficiency, sleep disturbances, use of sleeping medication, and daytime dysfunction. Each component is scored from 0 to 3. The seven component scores are summed to produce a global PSQI score ranging from 0 to 21, with higher scores indicating poorer habitual sleep quality. The original validation study demonstrated satisfactory internal consistency, test–retest reliability, and validity in distinguishing individuals with and without clinically relevant sleep difficulties [11]. A global score greater than 5 was used descriptively to distinguish participants reporting poor sleep quality from those reporting good sleep quality. The primary analyses used the continuous global PSQI score rather than the dichotomized classification.

#### 2.5.2. Insomnia Severity Index

The perceived severity of insomnia symptoms during the preceding two weeks was assessed using the seven-item ISI [12]. The instrument evaluates difficulties initiating and maintaining sleep, early-morning awakening, satisfaction with the current sleep pattern, interference with daytime functioning, noticeability of the sleep problem, and associated distress. Each item is rated from 0 to 4, producing a total score from 0 to 28. Higher scores indicate more severe insomnia symptoms. The ISI has demonstrated adequate internal consistency, concurrent validity, and sensitivity to changes in perceived insomnia severity [12]. For descriptive presentation, scores of 0–7 were classified as no clinically significant insomnia symptoms, 8–14 as subthreshold insomnia symptoms, 15–21 as moderate insomnia-severity range, and 22–28 as severe insomnia-severity range.

#### 2.5.3. Fatigue Severity Scale

The perceived influence of fatigue on functioning during the preceding week was assessed using the nine-item FSS [13]. The items address the influence of fatigue on motivation, physical functioning, sustained activity, responsibilities, work, and social life. Each statement is rated on a seven-point scale ranging from 1, indicating strong disagreement, to 7, indicating strong agreement. The nine responses were summed to produce a total score ranging from 9 to 63. Higher values indicate a greater perceived impact of fatigue. The original validation study demonstrated internal consistency, concurrent validity, and the ability to distinguish individuals with fatigue-related clinical conditions from healthy controls [13]. A total score of at least 36, which is equivalent to an average item score of at least 4, was used descriptively to identify elevated fatigue. The continuous total FSS score was used in the inferential analyses.

### 2.6. Countermovement Jump Assessment

Countermovement jump (CMJ) height was assessed using the OptoJump photoelectric measurement system (Microgate, Bolzano, Italy). The system determines flight time using transmitting and receiving bars positioned above the floor and calculates vertical jump height from the duration of the flight phase. OptoJump has demonstrated strong concurrent validity and excellent test–retest reliability for the assessment of vertical jump height [19].

Jump height was calculated as follows:

Jump height (cm) = [9.81 × flight time^2^ (s^2^)/8] × 100

Before testing, participants completed a standardized warm-up consisting of 5 min of light running at a self-selected intensity, dynamic mobilization of the lower-limb joints, 10 bodyweight squats, and five progressive CMJs performed with increasing effort. The final two practice jumps were performed at near-maximal intensity. These practice attempts were used to familiarize participants with the required technique and were not included in the analysis.

Each participant subsequently performed six separate maximal CMJs at 0, 2, 4, 6, 8, and 10 min, providing a standardized 2 min recovery interval between consecutive trials. One valid maximal jump was recorded at each time point. Participants began each trial in an upright position with their feet approximately shoulder-width apart and their body mass distributed evenly between both lower limbs. Their hands remained on their hips throughout the movement to eliminate the contribution of the arm swing.

Participants performed a rapid countermovement to a self-selected depth, followed immediately by a maximal vertical jump. They were instructed to take off simultaneously from both feet, maintain extension of the hips and knees during the flight phase, and land simultaneously on both feet within the measurement area. Participants were encouraged to use a consistent countermovement depth and landing technique across all trials.

All tests were performed in athletic footwear on a level, non-slip indoor surface. Standardized verbal encouragement was provided before each attempt. A trial was considered invalid if the participant removed either hand from the hips, visibly flexed the hips or knees during flight, took off, or landed asymmetrically, landed outside the measurement area, or lost balance during landing. Invalid attempts were repeated after sufficient recovery while maintaining the standardized testing schedule. All CMJ assessments were supervised by the same experienced investigator to ensure consistent instructions and evaluation of jump technique.

### 2.7. Derived Countermovement Jump Outcomes

Mean CMJ height was calculated as the arithmetic mean of the six trials. Best CMJ height was defined as the highest value obtained across the six trials. Within-participant variability was initially expressed as the standard deviation of the six measurements. The conventional within-participant coefficient of variation was calculated as

CV (%) = (within-participant SD/mean CMJ height) × 100

Because the conventional CV may include both random trial-to-trial variation and systematic improvement or deterioration over time, a trend-adjusted CV was also calculated. For each participant, a simple linear regression was fitted using CMJ height as the dependent variable and the actual measurement time points as the independent variable.

The residual standard error was calculated as the square root of the sum of squared residuals divided by n − 2, where n was equal to six trials.

The trend-adjusted CV was subsequently calculated as

Trend-adjusted CV (%) = (residual standard error/mean CMJ height) × 100

The percentage change between the first and final trials was calculated as

First-to-last change (%) = [(CMJ at 10 min − CMJ at 0 min)/CMJ at 0 min] × 100

Positive values indicate that the final CMJ was higher than the initial CMJ, whereas negative values indicate a lower final result.

An individual CMJ slope was calculated for each participant using the following model:

*CMJ_i_* = *β*_0_ + *β*_1_*t_i_* + *ε_i_*

where CMJ_i_ represents jump height during trial i, t_i_ represents the corresponding measurement time in minutes, β_0_ represents the individual intercept, β_1_ represents the individual linear performance slope, and ε_i_ represents the residual error. The individual slope was expressed in cm/min. A positive value indicates an upward CMJ trajectory, whereas a negative value indicates a downward trajectory.

### 2.8. Statistical Analysis

Statistical analyses were conducted using Python, version 3.13.5. NumPy, version 2.3.5 [20], and SciPy, version 1.17.0 [21], were used for data management and numerical calculations. Regression and linear mixed-effects models were estimated using statsmodels, version 0.14.6 [22]. Continuous variables are presented as mean ± standard deviation and 95% confidence interval, whereas categorical variables are reported as frequencies and percentages. Data distributions were evaluated using the Shapiro–Wilk test and visual inspection of histograms and quantile–quantile plots. Associations among PSQI, ISI, and FSS scores were assessed using Spearman’s rank correlation coefficients.

#### 2.8.1. Consistency of Countermovement Jump Performance

Relative consistency across the six CMJ trials was assessed using intraclass correlation coefficients based on a two-way mixed-effects model with a consistency definition [23]. ICC(3,1) represented the consistency of a single CMJ trial, whereas ICC(3,k) represented the consistency of the mean of the six trials. Within-participant variability was described using the conventional coefficient of variation and the trend-adjusted coefficient of variation. Absolute within-session measurement error was summarized using the standard error of measurement (SEM), equivalent here to the typical error, calculated as the square root of the residual mean square from the ICC model. The 95% minimal detectable change (MDC95) for the difference between two single CMJ measurements was calculated as SEM × 1.96 × √2. A distribution-based smallest worthwhile change was calculated as 0.2 of the between-participant standard deviation of participant mean CMJ height. Because all six trials were obtained within a single session and could include genuine time-related changes in performance, these indices were treated as descriptive benchmarks for practical interpretation rather than as an independent test–retest validation.

#### 2.8.2. Linear Mixed-Effects Models

The primary analyses were performed using linear mixed-effects models including all six CMJ measurements recorded at 0, 2, 4, 6, 8, and 10 min. Separate models were estimated for PSQI, ISI, and FSS. CMJ height was entered as the dependent variable. Each model included measurement time, the standardized questionnaire score, the time × questionnaire interaction, sex, age, and BMI as fixed effects. A participant-specific random intercept was included to account for the clustering of repeated observations within participants and between-participant differences in average CMJ height. The primary estimand was the population-average association between questionnaire score and linear within-session change, represented by the fixed time × questionnaire interaction. Time was entered as a continuous variable using the actual measurement times and was centred around its mean. Questionnaire scores, age, and BMI were standardized before analysis. A negative time × questionnaire coefficient represented a less favourable CMJ trajectory at higher questionnaire values.

Model robustness was examined using several complementary sensitivity analyses. First, the PSQI model was re-estimated with correlated participant-specific random intercepts and random slopes for centred time. Maximum-likelihood versions of the random-intercept and random-slope models were compared using the Akaike and Bayesian information criteria. Time was then treated as a categorical variable, with the overall time × PSQI interaction evaluated using a five-degree-of-freedom Wald test and the corresponding linear-trend contrast estimated separately. A model including linear and quadratic time terms and their interactions with PSQI was also fitted. Conditional residuals from the random-slope model were evaluated using the Shapiro–Wilk test, residual skewness and excess kurtosis, the association between absolute residuals and fitted values, and the Brown–Forsythe test across measurement times. Participant influence was examined by repeating the analysis after sequentially excluding each participant. Because the diagnostics indicated departures from normality and homogeneous residual variance, the adjusted participant-level slope analysis was additionally estimated using HC3 heteroscedasticity-consistent standard errors and a participant-level bootstrap with 10,000 resamples.

#### 2.8.3. Participant-Level Regression Models

Secondary multiple linear regression models examined the associations of PSQI, ISI, and FSS with mean CMJ height, best CMJ height, conventional coefficient of variation, trend-adjusted coefficient of variation, first-to-last percentage change, and individual CMJ slope. A separate model was estimated for each questionnaire and CMJ outcome. All models were adjusted for sex, age, and BMI. Questionnaire predictors were standardized, whereas the outcomes remained in their original units. Additional models for first-to-last change and individual CMJ slope included PSQI, ISI, and FSS simultaneously. The first-to-last change and individual CMJ slope analyses were secondary participant-level summaries derived from the same six CMJ measurements used in the primary mixed-effects models. They were included to provide alternative descriptions of the within-session trajectory and were not treated as statistically independent confirmations or replications of the mixed-model interaction.

#### 2.8.4. Control of Multiple Testing

The Benjamini–Hochberg false-discovery-rate procedure was used to account for multiple testing [24]. The three time × questionnaire interactions constituted the primary multiplicity family. The six analyses examining first-to-last change and individual CMJ slope for PSQI, ISI, and FSS constituted the trajectory-focused secondary family. For transparency, an additional correction was applied across all 18 questionnaire–outcome regression models, including mean CMJ height, best CMJ height, conventional CV, trend-adjusted CV, first-to-last change, and individual CMJ slope. Unstandardized regression coefficients, 95% confidence intervals, unadjusted *p* values, and false-discovery-rate-adjusted q values are reported. Statistical significance was defined as *p* < 0.05 for unadjusted analyses and q < 0.05 after correction within the relevant multiplicity family.

## 3. Results

### 3.1. Participant Characteristics

Participant characteristics are presented in Table 1. The mean PSQI, ISI, and FSS scores were 5.6 ± 2.7, 7.9 ± 4.6, and 28.9 ± 11.3, respectively. Poor sleep quality (PSQI > 5) was identified in 60 participants (50.8%), insomnia symptoms (ISI ≥ 8) in 63 participants (53.4%), and an elevated FSS score (FSS ≥ 36) in 38 participants (32.2%). Based on the ISI categories, 55 participants (46.6%) had no clinically significant insomnia, 50 (42.4%) had subthreshold insomnia, and 13 (11.0%) had scores within the moderate insomnia-severity range; no participant had severe insomnia symptoms. The questionnaire scores were moderately interrelated: PSQI correlated with ISI (Spearman’s ρ = 0.565, *p* < 0.001) and FSS (ρ = 0.477, *p* < 0.001), while ISI correlated with FSS (ρ = 0.435, *p* < 0.001).

### 3.2. Repeated CMJ Performance and Repeatability

Descriptive statistics for the six CMJ trials are shown in Table 2. Mean CMJ height across all trials was 34.0 ± 7.3 cm (95% CI 32.6–35.3), and the best recorded CMJ height was 35.9 ± 7.4 cm (95% CI 34.5–37.2). The average first-to-last change was 2.0 ± 6.9% (95% CI 0.7–3.2). Repeated CMJ performance showed high relative consistency. The single-trial ICC(3,1) was 0.943, while the reliability of the mean of six trials, ICC(3,k), was 0.990. Mean within-participant CV was 4.8 ± 2.6%, and mean trend-adjusted CV was 4.5 ± 2.7%. The within-session SEM/typical error was 1.79 cm, corresponding to an MDC95 of 4.96 cm for the difference between two single-trial measurements. The distribution-based smallest worthwhile change was 1.46 cm.

### 3.3. Associations of Sleep Quality, Insomnia Symptoms, and Fatigue with Repeated CMJ Performance

In the adjusted linear mixed-effects models, PSQI was not associated with the average CMJ level across trials (B = 0.088 cm per 1-SD higher PSQI, 95% CI −0.927 to 1.103, *p* = 0.865). However, the time × PSQI interaction was significant (B = −0.061 cm/min per 1-SD higher PSQI, 95% CI −0.099 to −0.023, *p* = 0.002, q = 0.005), indicating that a higher PSQI score was associated with a smaller increase in CMJ height across repeated trials. At the mean PSQI score, the estimated CMJ time slope was positive (B = 0.060 cm/min, 95% CI 0.022 to 0.098, *p* = 0.002). In the sensitivity model including correlated participant-specific random intercepts and random slopes for time, the estimated standard deviation of the random slopes was 0.096 cm/min. The time × PSQI interaction remained virtually unchanged and statistically significant (B = −0.062 cm/min per 1-SD higher PSQI, 95% CI −0.103 to −0.020, *p* = 0.004), indicating that the primary finding was not dependent on the random-intercept-only specification. This model-based estimate represents residual between-participant heterogeneity in time slopes after accounting for the fixed effects and therefore differs from the unadjusted standard deviation of 0.236 cm/min calculated from slopes estimated separately for each participant. Neither ISI nor FSS was associated with the average CMJ level, and their interactions with time were not significant (Table 3). Sex was associated with CMJ height in all three mixed models, with men demonstrating an adjusted mean CMJ height approximately 11.1–11.2 cm greater than women (all *p* < 0.001). Age and BMI were not associated with CMJ height in these models.

The participant-level regression models provided exploratory alternative summaries of the same six CMJ measurements evaluated in the mixed-effects models (Table 4). PSQI was not associated with mean CMJ height, best CMJ height, conventional CV, or trend-adjusted CV (all *p* ≥ 0.868). In contrast, each 1-SD higher PSQI score was associated with a 1.62-percentage-point smaller first-to-last change (95% CI −2.86 to −0.38, *p* = 0.011) and a 0.063 cm/min smaller individual CMJ slope (95% CI −0.104 to −0.021, *p* = 0.004). These associations remained significant when the six trajectory-focused analyses were treated as the multiplicity family (q = 0.032 and q = 0.023, respectively), but not when all 18 participant-level regressions were treated as a single family (q = 0.096 and q = 0.068, respectively). Because first-to-last change and individual slope were derived from the same six trials included in the mixed-effects model, these exploratory results represent dependent alternative summaries rather than independent confirmations or replications of the PSQI × time interaction. When PSQI, ISI, and FSS were entered simultaneously, PSQI was associated with first-to-last change and individual CMJ slope, whereas ISI and FSS were not associated with either outcome (Table 5).

All sensitivity models converged successfully. Comparison of the maximum-likelihood fits provided mixed evidence regarding the random-effects structure. AIC was slightly lower for the random-slope model than for the random-intercept model (3316.4 vs. 3319.4), whereas BIC favoured the more parsimonious random-intercept model (3360.4 vs. 3366.6). When time was treated as categorical, the five-degree-of-freedom omnibus time × PSQI interaction did not reach conventional statistical significance (χ^2^(5) = 10.48, *p* = 0.063). However, the corresponding linear-trend contrast remained significant (B = −0.061 cm/min per 1-SD higher PSQI, 95% CI −0.100 to −0.023, *p* = 0.002). In the quadratic model, the linear time × PSQI interaction remained significant (B = −0.061 cm/min, 95% CI −0.100 to −0.023, *p* = 0.002), whereas the time^2^ × PSQI interaction was not significant (B = 0.002 cm/min^2^, 95% CI −0.011 to 0.015, *p* = 0.722). Standardized conditional residuals departed from normality (Shapiro–Wilk W = 0.958, *p* < 0.001; skewness = −0.62; excess kurtosis = 2.95), and residual variance differed across measurement times (Brown–Forsythe F(5, 702) = 4.53, *p* < 0.001). However, absolute residual magnitude was not associated with fitted CMJ values (Spearman’s ρ = 0.061, *p* = 0.103). The adjusted association between PSQI and individual CMJ slope remained significant with HC3 standard errors (B = −0.063 cm/min, 95% CI −0.110 to −0.015, *p* = 0.011) and in the participant-level bootstrap analysis (10,000 resamples; 95% CI −0.108 to −0.013). Leave-one-participant-out estimates ranged from −0.068 to −0.049 cm/min, with all *p* ≤ 0.022.

## 4. Discussion

The study hypothesis was only partially supported. Higher PSQI scores were associated with a smaller increase in CMJ height across six trials, as indicated by the primary PSQI × time interaction, which remained significant after correction across the three questionnaire × time tests (q = 0.005). PSQI was not associated with mean CMJ height, best CMJ height, or within-participant variability, and ISI and FSS were unrelated to CMJ level or trajectory. Exploratory first-to-last change and individual slope analyses showed associations in the same direction, but these remained significant only within the six trajectory-focused analyses (q = 0.032 and q = 0.023) and not after correction across all 18 participant-level regressions (q = 0.096 and q = 0.068). Because these outcomes were derived from the same six measurements, they are dependent alternative summaries rather than independent confirmations of the primary result.

The absence of associations with mean or best CMJ height is consistent with mixed evidence regarding brief maximal exercise. Cullen et al. [25] reported reduced CMJ performance after complete and partial sleep deprivation, whereas other studies have found preserved jump height despite impairments in more prolonged or cognitively demanding tasks [7]. Importantly, the PSQI reflects habitual sleep quality during the preceding month and is not equivalent to experimentally imposed sleep deprivation or sleep obtained on the night immediately before testing. Accordingly, the PSQI score cannot be interpreted as an indicator of the participant’s acute physiological state during the CMJ session [11].

The distinction between best and repeated performance remains relevant. Claudino et al. [26] suggested that average CMJ performance may be more sensitive than the best attempt for monitoring neuromuscular status. In the present study, however, PSQI was unrelated to both measures; the association concerned only the direction of change across trials and should not be interpreted as a general reduction in maximal performance. CMJ height increased modestly across the six trials. The negative PSQI × time interaction therefore indicates a smaller increase, not a progressive fatigue-related decline or evidence that participants with poorer sleep benefited less from a measured performance-optimization process. Each 1-SD higher PSQI score corresponded to an approximately 0.61 cm smaller estimated change over 10 min (95% CI 0.23–0.99 cm in magnitude). Although statistically significant, this association was small and its practical importance is uncertain. Familiarization, continued task-specific warm-up, technical adjustment, and changes in arousal or readiness are possible explanations for the overall positive trajectory, but none was measured; consequently, no specific mechanism can be inferred. Jump height derived from flight time may not detect changes in movement strategy. Previous studies have identified fatigue- or recovery-related alterations in eccentric and concentric phase characteristics even when jump height or other conventional outcomes were preserved [27,28,29,30,31]. Because the present study did not assess ground-reaction force, impulse, movement duration, countermovement depth, or interlimb asymmetry, it cannot determine whether sleep quality was associated with such mechanical changes. PSQI was also unrelated to conventional or trend-adjusted within-participant CV. Thus, the observed association concerned systematic change rather than random dispersion between trials. Variability and trajectory describe different features of repeated performance: a participant may show little dispersion around the mean while still exhibiting a progressive increase or decrease [32]. The measurement-error estimates further limit practical interpretation. The 0.61 cm difference was smaller than the within-session SEM/typical error of 1.79 cm, the distribution-based smallest worthwhile change of 1.46 cm, and the MDC95 of 4.96 cm for two single-trial measurements. Absolute error for an individual measurement differs from the sampling uncertainty of a population-level regression coefficient, so a smaller association may still be statistically detectable across repeated observations. Nevertheless, the result does not support individual diagnosis or a practical decision threshold. Monitoring reviews likewise emphasize measurement error, responsiveness, and contextual interpretation [28,33].

The null ISI and FSS findings may reflect differences in constructs and recall periods. The ISI concerns insomnia symptoms over two weeks, whereas the FSS assesses the functional impact of fatigue over the previous week rather than acute neuromuscular fatigue. Subjective indicators and objective performance measures may capture different aspects of recovery and need not correlate consistently [32,33]. Restricted symptom severity may also have contributed. Most participants had no clinically significant or only subthreshold insomnia, none had severe clinical insomnia, and approximately one-third had an FSS score of at least 36. Mild complaints may not be reflected during a brief, highly motivated maximal task, although this possibility was not directly tested. The PSQI is frequently used to monitor sleep in athletes [34,35], but it was developed as a composite clinical screening measure rather than an acute readiness tool. Its seven heterogeneous components further limit interpretation of the global score. Psychometric evidence in athletes also supports caution. Tan et al. [36] reported a multidimensional PSQI structure and suggested that the one-month response period may be disproportionately influenced by recent sleep experiences. The present finding should therefore be interpreted as an association with composite, habitual self-reported sleep quality, not with a specific or objectively verified sleep deficiency. The small effect, its relation to within-session error estimates, and the lack of validation against established recovery markers preclude using PSQI or CMJ trajectory alone to diagnose sleep disturbance or guide readiness and warm-up decisions. Longitudinal, within-person monitoring that combines subjective, training-load, and performance data may be more informative, but this application requires validation [33,34,35].

Recent evidence further supports separating habitual sleep–performance associations from the effects of acute sleep deprivation. Objectively measured sleep duration and efficiency were associated with longitudinal improvements in peak power in collegiate baseball players [37], while individualized sleep-hygiene education improved CMJ performance in female footballers [38]. Conversely, a recent meta-analysis indicated that sleep deprivation impaired explosive power, although effects varied across performance domains and protocols [39]. These findings support longitudinal, multimodal assessment rather than reliance on a single retrospective questionnaire.

Habitual sleep-quality questionnaires should not be used as stand-alone indicators of acute CMJ readiness. However, the present findings suggest that repeated trials may provide information not captured by a single best or mean performance value, although the small observed association and its uncertain measurement precision preclude individual-level recommendations. Future longitudinal and experimental studies should combine subjective and objective sleep measures with standardized CMJ familiarization, repeated reliability assessment, and direct evaluation of potential physiological and behavioural mechanisms. Such studies should determine whether within-session CMJ trajectories are reproducible, sensitive to changes in sleep, and practically meaningful in athletic monitoring.

Study strengths include the comparatively large sample of resistance-trained adults, six maximal CMJ trials, mixed-effects modelling of the complete repeated-measures structure, and analysis of all questionnaire scores as continuous variables. Several limitations remain. The cross-sectional design precludes causal inference, and the PSQI assessed the preceding month whereas CMJ performance was measured on one day; sleep duration and quality on the preceding night were unknown. Sleep was assessed only by retrospective self-report, without actigraphy, polysomnography, or prospective diaries, and the questionnaires had different recall periods. Recent training load, muscle soreness, chronotype, psychological stress, and menstrual-cycle status were also not assessed. Testing occurred between 09:00 and 12:00, but exact individual testing time was not included, and adherence to caffeine and alcohol restrictions was not objectively verified. Because these unmeasured or incompletely verified factors may have been associated with both sleep-related questionnaire scores and CMJ performance, their omission represents potential residual confounding. Although age was included as a continuous covariate, the 18–32-year range may have introduced residual age-related heterogeneity. Women represented less than one-quarter of the sample. Although sex was included as a covariate, the study was not designed to evaluate sex-specific associations; consequently, the findings apply to the combined sample and cannot establish equivalent associations in women and men. The photoelectric system did not provide force–time variables, and no independent repeated-session reliability protocol was conducted; the systematic increase across the six trials also prevents these trials from providing a pure estimate of random measurement error. The processes underlying this increase were not measured. Finally, the exploratory first-to-last and slope analyses were statistically dependent on the primary model and lost significance when all 18 participant-level regressions were corrected together. Future studies should combine repeated-day CMJ testing with sleep diaries, actigraphy, recent training-load data, and force-platform analysis. Experimental sleep interventions and independent reliability assessments are needed to test causality, identify potential mechanisms, and establish measurement-informed thresholds before CMJ trajectory can be used in recovery-related decisions.

## 5. Conclusions

Habitual sleep quality was not associated with average CMJ height, the best CMJ result, or within-participant variability in resistance-trained adults. However, poorer PSQI scores were associated with an attenuated improvement and a less favourable performance trajectory across successive maximal CMJ trials. Insomnia severity and perceived fatigue were not associated with the level, variability, or trajectory of CMJ performance. These findings apply to the combined sample, therefore sex-specific associations were not evaluated and should not be inferred.

The results suggest that habitual sleep quality may be related more closely to systematic changes across repeated maximal attempts than to the capacity to produce a single maximal effort. Analysing several standardized CMJ trials may therefore provide complementary information for CMJ within-session performance patterns, but the small associations observed do not establish causality and should not be interpreted as evidence of fatigue resistance, recovery status, neuromuscular readiness, clinical sleep impairment, or increased injury risk. Although CMJ trajectory could ultimately complement subjective recovery indicators, recent training exposure, and other objective measures, the trajectory observed in the present protocol remains exploratory and should not yet be used as an isolated decision-making tool. Validation across repeated sessions and against direct measures of sleep, fatigue, and movement mechanics is required before any applied use.

## Figures and Tables

**Table 1:** Participant characteristics and questionnaire scores.

Characteristic	Total (n = 118)	Men (n = 90)	Women (n = 28)
Age (years)	20.9 ± 2.9 (20.4–21.4)	21.1 ± 3.2 (20.4–21.7)	20.4 ± 1.9 (19.6–21.1)
Body height (cm)	177.1 ± 8.9 (175.5–178.8)	179.8 ± 7.9 (178.1–181.5)	168.6 ± 5.9 (166.3–170.9)
Body mass (kg)	74.3 ± 12.6 (72.0–76.6)	78.4 ± 10.7 (76.1–80.6)	61.3 ± 9.3 (57.7–64.9)
BMI (kg/m^2^)	23.6 ± 3.1 (23.0–24.2)	24.2 ± 2.9 (23.6–24.8)	21.6 ± 3.1 (20.4–22.7)
PSQI score	5.6 ± 2.7 (5.1–6.1)	5.6 ± 2.7 (5.0–6.2)	5.7 ± 2.7 (4.6–6.7)
ISI score	7.9 ± 4.6 (7.1–8.7)	8.2 ± 4.7 (7.2–9.2)	7.1 ± 4.2 (5.5–8.7)
FSS score	28.9 ± 11.3 (26.9–31.0)	28.5 ± 11.7 (26.1–31.0)	30.1 ± 9.7 (26.4–33.9)
PSQI > 5, n (%)	60 (50.8)	44 (48.9)	16 (57.1)
ISI ≥ 8, n (%)	63 (53.4)	49 (54.4)	14 (50.0)
FSS ≥ 36, n (%)	38 (32.2)	29 (32.2)	9 (32.1)

Data are presented as mean ± standard deviation (95% confidence interval) unless otherwise indicated. BMI, body mass index; FSS, Fatigue Severity Scale; ISI, Insomnia Severity Index; PSQI, Pittsburgh Sleep Quality Index.

**Table 2:** Descriptive statistics and consistency of countermovement jump performance.

Outcome	Mean ± SD (95% CI) or Estimate
CMJ at 0 min (cm)	33.7 ± 7.2 (32.4–35.0)
CMJ at 2 min (cm)	33.7 ± 7.4 (32.3–35.0)
CMJ at 4 min (cm)	34.0 ± 7.4 (32.7–35.4)
CMJ at 6 min (cm)	33.9 ± 7.7 (32.5–35.3)
CMJ at 8 min (cm)	34.0 ± 7.6 (32.6–35.4)
CMJ at 10 min (cm)	34.4 ± 7.7 (33.0–35.8)
Mean CMJ height (cm)	34.0 ± 7.3 (32.6–35.3)
Best CMJ height (cm)	35.9 ± 7.4 (34.5–37.2)
Within-participant CV (%)	4.8 ± 2.6 (4.3–5.2)
Trend-adjusted CV (%)	4.5 ± 2.7 (4.0–5.0)
SEM/typical error (cm)	1.79
MDC95 for two single-trial measurements (cm)	4.96
Smallest worthwhile change (cm)	1.46
First-to-last change (%)	2.0 ± 6.9 (0.7–3.2)
Individual CMJ slope (cm/min)	0.060 ± 0.236 (0.017–0.103)
ICC(3,1)	0.943
ICC(3,k), k = 6	0.990

N = 118. CMJ, countermovement jump; CV, coefficient of variation; ICC, intraclass correlation coefficient. ICC(3,1) reflects single-trial relative consistency, whereas ICC(3,k) reflects the consistency of the mean of six trials. CMJ trials were performed at 0, 2, 4, 6, 8, and 10 min. SEM, standard error of measurement; MDC95, 95% minimal detectable change. MDC95 was calculated as SEM × 1.96 × √2. The smallest worthwhile change was calculated as 0.2 of the between-participant standard deviation of participant mean CMJ height.

**Table 3:** Adjusted linear mixed-effects models for repeated countermovement jump height.

Questionnaire	Main Effect, B (95% CI)	*p*	Time × Questionnaire, B (95% CI)	*p*	q
PSQI	0.088 (−0.927 to 1.103)	0.865	−0.061 (−0.099 to −0.023)	0.002	0.005
ISI	0.372 (−0.649 to 1.393)	0.475	−0.001 (−0.040 to 0.037)	0.954	0.954
FSS	0.079 (−0.935 to 1.093)	0.879	−0.030 (−0.069 to 0.008)	0.124	0.186

B values are unstandardized coefficients. Questionnaire scores, age, and BMI were standardized. Models included continuous centred time, questionnaire score, time × questionnaire interaction, sex, age, BMI, and a participant-specific random intercept. q values are Benjamini–Hochberg adjusted across the three time × questionnaire interactions.

**Table 4:** Adjusted participant-level regression models for countermovement jump outcomes.

Outcome	Predictor	B per 1 SD (95% CI)	*p*	q	q (Trajectory Family)	ΔR^2^
Mean CMJ height (cm)	PSQI	0.09 (−0.96 to 1.14)	0.868	0.940		0.000
	ISI	0.37 (−0.68 to 1.43)	0.486	0.940		0.003
	FSS	0.08 (−0.97 to 1.13)	0.882	0.940		0.000
Best CMJ height (cm)	PSQI	0.05 (−1.01 to 1.11)	0.926	0.940		0.000
	ISI	0.30 (−0.77 to 1.37)	0.580	0.940		0.002
	FSS	0.05 (−1.01 to 1.11)	0.930	0.940		0.000
Within-participant CV (%)	PSQI	−0.02 (−0.50 to 0.46)	0.940	0.940		0.000
	ISI	−0.29 (−0.77 to 0.19)	0.233	0.838		0.012
	FSS	−0.14 (−0.62 to 0.33)	0.551	0.940		0.003
Trend-adjusted CV (%)	PSQI	−0.03 (−0.52 to 0.46)	0.917	0.940		0.000
	ISI	−0.26 (−0.76 to 0.23)	0.290	0.870		0.010
	FSS	−0.08 (−0.56 to 0.41)	0.762	0.940		0.001
First-to-last change (%)	PSQI	−1.62 (−2.86 to −0.38)	0.011	0.096	0.032	0.056
	ISI	−0.53 (−1.80 to 0.75)	0.418	0.940	0.501	0.006
	FSS	−1.02 (−2.28 to 0.23)	0.110	0.657	0.219	0.022
Individual CMJ slope (cm/min)	PSQI	−0.063 (−0.104 to −0.021)	0.004	0.068	0.023	0.070
	ISI	−0.006 (−0.050 to 0.038)	0.784	0.940	0.784	0.001
	FSS	−0.029 (−0.072 to 0.015)	0.193	0.838	0.289	0.015

All models were adjusted for sex, age, and BMI. B represents the adjusted outcome difference associated with a 1-SD higher questionnaire score. q (18 tests) applies the Benjamini–Hochberg procedure across all 18 questionnaire–outcome models. q (trajectory family) applies only to the six models for first-to-last change and individual CMJ slope. ΔR^2^ represents incremental variance explained beyond the covariates.

**Table 5:** Simultaneous regression models for CMJ trajectory outcomes.

Outcome	Predictor	B per 1 SD (95% CI)	*p*	Model R^2^
First-to-last change (%)	PSQI	−1.83 (−3.42 to −0.23)	0.025	0.071
	ISI	0.76 (−0.82 to 2.34)	0.341	
	FSS	−0.50 (−1.94 to 0.94)	0.494	
Individual CMJ slope (cm/min)	PSQI	−0.086 (−0.140 to −0.033)	0.002	0.119
	ISI	0.048 (−0.005 to 0.101)	0.073	
	FSS	−0.009 (−0.057 to 0.039)	0.712	

Models included PSQI, ISI, and FSS simultaneously and were adjusted for sex, age, and BMI. B represents the adjusted outcome difference associated with a 1-SD higher questionnaire score while holding the other questionnaire scores and covariates constant.

## Data Availability

The data presented in this study are available on request from the author. The data are not publicly available due to privacy and ethical considerations related to participant data.
